# Extremely delayed solitary cerebral metastasis in patient with T1N0M0 renal cell carcinoma after radical nephrectomy: Case report and literature review

**DOI:** 10.1097/MD.0000000000025586

**Published:** 2021-04-16

**Authors:** Yoon-Hee Choo, Youngbeom Seo, Joonhyuk Choi

**Affiliations:** a Department of Neurosurgery; b Department of Pathology, Yeungnam University Hospital, Yeungnam University College of Medicine Daegu, Republic of Korea.

**Keywords:** brain, delayed, late, metastasis, nephrectomy, renal cell carcinoma

## Abstract

**Rationale:**

Although renal cell carcinoma (RCC) is one of the common origins of brain metastasis, few cases of extremely delayed brain metastasis from RCC, more than 10 years after nephrectomy, have been reported. We present a rare case of extremely delayed brain metastasis from RCC, also performed a literature review to increase knowledge of the characteristics for extremely delayed brain metastasis from RCC.

**Patient concerns:**

A 72-year-old man presented with right-sided hemiplegia and dysarthria. The patient had a history of radical nephrectomy for RCC with stage T1N0M0 15 years earlier.

**Diagnosis:**

Magnetic resonance imaging with contrast revealed a 2-cm sized non-homogenous enhanced mass in the left frontal lobe with peritumoral edema. The pathological examination after surgery reported metastatic clear cell RCC.

**Interventions:**

A craniotomy for removal of the mass was performed at the time of diagnosis. Stereotactic radiosurgery was performed for the tumor bed 3 weeks after craniotomy, and then, chemotherapy was started 2 months after the SRS.

**Outcomes:**

Metastasis progressed to multiple organs 6 months after the craniotomy. The patient chose a hospice and no longer visited the hospital.

**Lessons:**

In cases with a history of nephrectomy for RCC, long period follow-up is necessary for monitoring RCC brain metastasis and pathologic diagnosis should be confirmed.

## 1 Introduction

Renal cell carcinoma (RCC) is the most common kidney cancer with an incidence of 2% to 3% of all malignant cancers in adults.^[1]^ It is observed that extremely delayed distant metastasis that occurs in other organs including the lung, bone, and liver 10 years after nephrectomy for RCC is not rare, with a prevalence of 4.7% to 11%.^[2]^ However, few cases of extremely delayed metastasis to the brain, occurring more than 10 years after the initial diagnosis of RCC, have been reported,^[2–17]^ and the mechanism of delayed metastasis is not clearly known. Here, we report a case of extremely delayed solitary brain metastasis of RCC with lymph node metastasis that occurred 15 years after nephrectomy.

## 2 Case presentation

**Figure 1:**
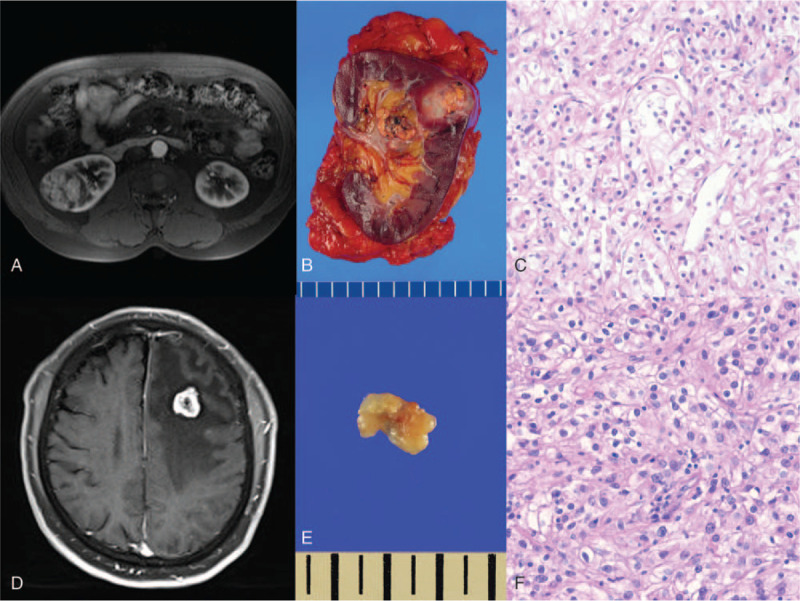
Preoperative magnetic resonance imaging (MRI) images and photomicrographs of specimens from renal cell carcinoma and brain metastasis. (A) The kidney MRI image of the preoperative renal cell carcinoma shows an approximately 6 cm-sized tumor in the right kidney. (B) Section of specimen obtained after nephrectomy 15 yr ago. (C) Photomicrograph of renal cell carcinoma stained with Hematoxylin and eosin (H&E) characterized by abundant clear cytoplasm (×100). (D) Gadolinium contrast enhanced T1-weighted MRI image shows a 2 cm-sized non-homogeneous enhanced and round-shaped mass in the left frontal lobe with peritumoral edema. (E) Section of specimen obtained after craniotomy and removal of mass. (F) Photomicrograph of brain tumor stained with H&E characterized by clear cytoplasm (×200).

**Figure 2:**
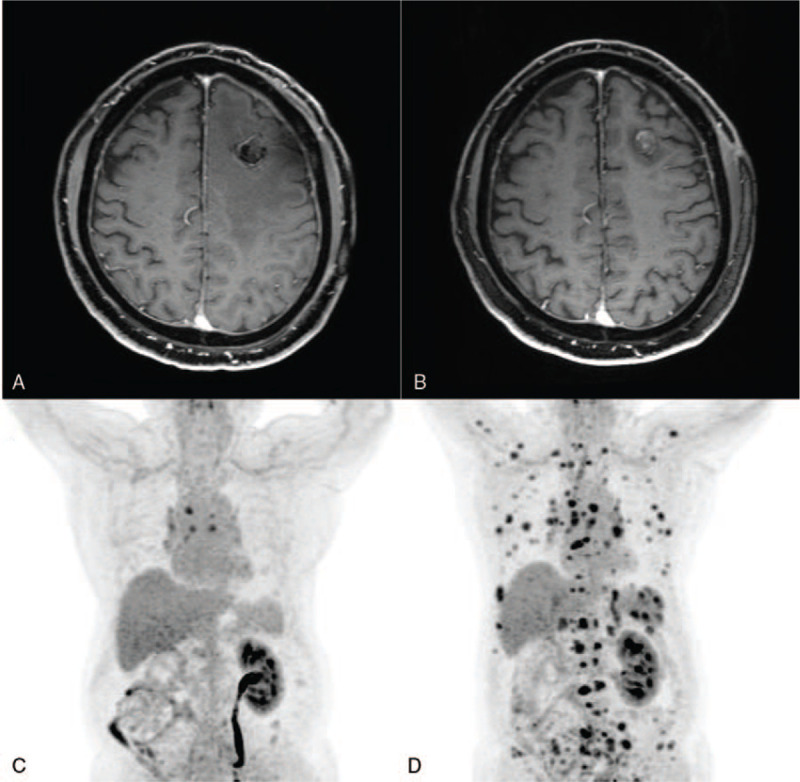
Series of magnetic resonance imaging (MRI) and whole-body positron emission tomography (PET) images. (A) Postoperative gadolinium-enhanced T1-weighted axial MRI performed a day after operation showing no evidence of remnant tumor. (B) Follow-up MRI at 4 mo after craniotomy. Gadolinium-enhanced T1-weighted axial MRI images showed no evidence of recurrence and decrease of peritumoral edema. (C) PET images taken at the time of diagnosis of brain metastasis. (D) Six months after surgery, follow-up PET was performed during chemotherapy. Multiple metastases were identified in the whole body.

A 72-year-old man presented with right-sided hemiparesis and dysarthria. Magnetic resonance imaging (MRI) with gadolinium showed a 2 cm-sized non-homogeneous enhanced and round-shaped mass in the left frontal lobe with peritumoral edema (Fig. 1D). Fifteen years previously, he had undergone a right radical nephrectomy for a 6 cm-sized mass on the kidney, following the histopathologic diagnosis of clear cell typed RCC (Fig. 1A–C). No evidence of metastasis was observed at that time (stage 1, T1, N0, M0), according to the tumor node metastasis (TNM) system, which is the most commonly used staging system established by the American Joint Committee on Cancer.^[18]^ A whole-body positron emission tomography (PET) study performed at the time of the current presentation showed a hypometabolic lesion in the left frontal lobe due to peritumoral edema, which was considered as brain metastasis, and several hypermetabolic lymph nodes at station 4R (right lower paratracheal nodes) and 7 (subcarinal nodes) (Fig. 2C).

The patient underwent brain surgery for histopathologic diagnosis and tumor removal. He underwent a frontal craniotomy and gross total resection of the tumor (Figs. 1E and 2A). The final histopathologic report revealed metastatic clear cell RCC with a Ki-67 index of 40%, which is primary in the kidney for both tumor and peritumoral tissue. Hematoxylin and eosin-stained tissue showed clear cytoplasm and round-to-oval-shaped nuclei (Fig. 1F). Three weeks after the craniotomy, stereotactic radiosurgery (SRS) was performed for the tumor bed because of the possibility of tumor cells based on the reports of the biopsy. There was no evidence of recurrence on a follow-up brain MRI performed 4 months after surgery (Fig. 2B). Two months after the SRS, he complained of chest pain in the right lateral side, and multiple bone metastases and left back muscle metastases were found on follow-up whole-body PET. Although chemotherapy was started at the oncology department, multiple distant metastases including the lung, liver, spleen, and adrenal gland were found 6 months after the operation (Fig. 2D). Subsequently, the patient chose a hospice and no longer visited the hospital.

## 3 Discussion

RCC is the most common kidney cancer with an incidence of 2% to 3% of all malignant cancers in adults.^[1]^ According to the TNM staging system, distant metastasis indicates difficulty in expecting a good prognosis.^[18]^ In fact, patients with stage IV RCC, distant metastatic RCC, had less than 10% of a 5-year survival with a median overall time of 6 to 10 months.^[18]^ Extremely delayed distant metastasis of RCC, over 10 years after nephrectomy, is not very rare, with a prevalence of 4.7% to 11%.^[2]^ The most common metastatic sites of RCC are the lungs, lymph nodes, bone, and liver.^[19,20]^

In comparison, brain metastasis occurs in 3.9% to 24% of patients with RCC and is most frequently detected within an average of 1 to 3 years after the nephrectomy.^[16,20]^ Similar to the cases of distant metastasis to other parts other than the brain, the prognosis of brain metastasis from RCC is poor; the median overall survival time after a diagnosis of brain metastasis was 10.7 months, and the 5-year survival rate was 12%.^[20]^ To the best of my knowledge, a total of 20 cases of extremely delayed brain metastases from RCC have been reported in English so far,^[2–17]^ making it a rare occurrence; all 21 cases including the present case are listed in Table 1. The cases summarized in Table 1 were searched by various combinations of search terms such as ‘brain or cerebral’, ‘late or delayed’, ‘metastasis or metastatic’, ‘renal or renal cell carcinoma’ and ‘nephrectomy’ in databases such as PubMed, Scholar Google, and Embase, and also referred to the lists summarized published paper. The median interval period from nephrectomy to brain metastasis diagnosis was 15 years (range, 11–26 years). Of the 21 patients, 17 had a solitary lesion and 4 had 2 to 3 multiple lesions diagnosed as brain metastasis.

**Table 1:** Summary of previously reported cases of extremely delayed brain metastasis from renal cell carcinoma.

Case	Author (yr)	Age	Sex	Interval^†^ (yr )	Solitary or multiple	Location	Diameter (mm)	TNM staging^‡^	Treatment	Survival period from the first brain surgery
1	Middleton^[3]^ (1967)	ND	M	14	Solitary	T	ND	ND	GTR	Alive 17 yr
2	Killebrew et al^[4]^ (1983)	55	F	13	Solitary	Lt. trigone	25	T2N?M0	GTR	Alive 4 yr
3	Ishikawa et al^[5]^ (1990)	46	F	14	Solitary	Lt. P	35^∗^	T?N?M0	GTR	Alive 28 mo
4	Ammirati et al^[6]^ (1993)	63	F	13	Solitary	Lt. CBLL	30^∗^	T?N0M0	GTR	Recurrence 9 mo; alive 18 mo after 2^nd^ craniotomy
5	Radley et al^[8]^ (1993)	78	M	18	Solitary	Lt. T	20	T?N?M0	GTR	Alive 17 yr
6	Radley et al^[8]^ (1993)	60	F	15	Solitary	Lt. T	ND	T?N?M0	GTR, RT	ND
7	Cervoni et al^[7]^ (1993)	61	M	13	Solitary	Rt. FR	ND	T?N0M0	GTR	Systemic spread after 53 mo and expired
8	Cervoni et al^[7]^ (1993)	65	F	17	Solitary	Rt. FR	ND	T?N0M0	GTR	Alive 58 mo
9	Jubelirer^[9]^ (1996)	86	F	15	Solitary	Lt. FR	ND	ND	STR	Decreased 6 weeks after craniotomy
10	Kuroki et al^[10]^ (1999)	86	F	12	Solitary	Lt. TP	30	ND	GTR, RT	ND
11	Kuroki et al^[10]^ (1999)	67	M	15	Multiple	Lt. FR	15	ND	GTR, RT	Alive 3 mo (Rt. P new lesion detected)
12	Roser et al^[11]^ (2002)	61	M	19	Solitary	Lt. FR	30^∗^	T1N0M0	GTR	Alive 14 mo (history of brain metastasis after 3 yr of nephrectomy)
13	Cimatti et al^[12]^ (2004)	67	M	26	Solitary	Rt. T	ND	T1N0M0	GTR, WBRT	Alive 36 mo
14	Cimatti et al^[12]^ (2004)	52	M	12	Multiple	Rt. P, Lt. CBLL	30, 8	ND	GTR, SRS	ND
15	Sadatomo et al^[13]^ (2005)	77	M	15	Solitary	Lt. trigone	20	T2N0M0	STR, GRS	Alive 7 mo
16	Montano et al^[14]^ (2007)	65	M	20	Solitary	Falx cerebri	20^∗^	ND	GTR	ND
17	Bademci et al^[15]^ (2008)	68	F	20	Solitary	Lt. TP	70^∗^	ND	GTR	ND (no recurrence for 4 mo)
18	Choi et al^[16]^ (2013)	76	F	18	Multiple	Vertex, 4^th^ ventricle	30^∗^, 10^∗^	ND	STR, GRS	Recurrence 4 yr, expire 6 yr
19	Aydin et al^[17]^ (2015)	72	M	11	Solitary	Rt. FP	80	ND	GTR	ND
20	Fukushima et al^[2]^ (2016)	60	M	22	Multiple	Rt. CBLL, Lt. P	27, 9.5	T1N0M0	GTR	Alive 36 mo
21	Present case	72	M	15	Solitary	Lt. FR	20	T1N0M0	GTR, SRS	Systemic spread 4 mo

RCC is considered to be radio- and chemo-resistant.^[20]^ Thus, surgical total resection is a standard treatment option for patients with brain metastases.^[20]^ Of the 21 patients with extremely delayed brain metastasis of RCC, 18 patients underwent total resection and 3 patients underwent subtotal resection. Although the total number of cases was not large (21 cases), the proportion of cases with a good prognosis was much higher in cases of total resection. In 2 cases, the patients expired: 1 patient underwent total resection for a solitary metastatic lesion and expired due to systemic spread,^[7]^ and the other underwent subtotal resection for multiple metastatic lesions and expired without systemic spread.^[16]^ We present a case of systemic metastasis accompanied by lymph node metastasis at the time of brain metastasis diagnosis, although total resection and SRS were performed for a single lesion. In addition, Fukushima et al^[2]^ reported that even in the case of multiple brain metastases, a good prognosis can be expected through total surgical resection. Total resection could be quite effective for the local control of extremely delayed brain metastatic lesions. In addition, SRS is also known to be effective in local metastatic tumor control.^[20]^

There are several hypotheses about the mechanism of extremely delayed metastasis after nephrectomy for RCC. First, it is probable that the dissemination of tumor cells occurred before the nephrectomy and grew slowly.^[19]^ Second, the microscopic metastatic lesions remain dormant for decades and begin to grow when the host immunopotency decreases. Several basic studies using rodent models have shown that single tumor cells spread to distant sites early on and have a period of dormancy.^[19]^

Histopathologic confirmation is essential for the diagnosis. Bademci et al^[15]^ and Montano et al^[14]^ reported a metastatic RCC mimicking meningioma, which was initially diagnosed as a meningioma on radiologic imaging tests. The histopathological characteristics of RCC, especially the clear cell type which occupy the RCC, are clear cytoplasm with a high lipid content during histological preparation.^[1]^ In some reports, the MiB-1 labeling index was less than 1%^[2,11]^ or 7%^[13]^ as a cell proliferation marker, and in this case, the cell proliferation rate was comparatively high, with a Ki-67 index of 40%. In the present case, the progression of the systemic spread of RCC after the first diagnosis of metastasis was relatively fast compared to that in previously reported cases of extremely delayed brain metastases of RCC. Cell proliferation is thought to be related to the prognosis of metastatic RCC.

## 4 Conclusion

We report a rare case of extremely delayed brain metastasis from RCC. If there is a history of RCC, it is necessary to conduct long-term follow-up for systemic metastasis. Furthermore, it is essential to suspect the metastasis of RCC and to confirm the diagnosis through pathologic examination.

## Author contributions

**Conceptualization:** Yoon-Hee Choo, Youngbeom Seo.

**Data curation:** Yoon-Hee Choo, Joonhyuk Choi.

**Investigation:** Yoon-Hee Choo.

**Methodology:** Youngbeom Seo.

**Writing – original draft:** Yoon-Hee Choo, Youngbeom Seo.

**Writing – review & editing:** Youngbeom Seo, Joonhyuk Choi.
